# Effect of antioxidant supplementation on the auditory threshold in sensorineural hearing loss: a meta-analysis^☆^

**DOI:** 10.1016/j.bjorl.2017.07.011

**Published:** 2017-08-26

**Authors:** Maria Eduarda Di Cavalcanti Alves de Souza, Klinger Vagner Teixeira da Costa, Paulo Augusto Vitorino, Nassib Bezerra Bueno, Pedro de Lemos Menezes

**Affiliations:** a Universidade Federal de Alagoas (UFAL), Rede Nordeste de Biotecnologia (RENORBIO), Biotecnologia em Saúde, Maceió, AL, Brazil; b Universidade Estadual de Ciências da Saúde de Alagoas (UNCISAL), Laboratório de Audição e Tecnologia (LATEC), Maceió, AL, Brazil; c Universidade Federal de Alagoas (UFAL), Maceió, AL, Brazil; d Universidade Estadual de Ciências da Saúde de Alagoas (UNCISAL), Maceió, AL, Brazil

**Keywords:** Hearing, Reactive oxygen species, Free radicals, Audição, Espécies de oxigênio reativas, Radicais livres

## Abstract

**Introduction:**

Hearing loss is conceptualized as any impairment of the ability to hear and/or detect speech or environment sounds, regardless of cause, type, or degree. It may occur at different stages of life; during pregnancy or childbirth, in childhood, adulthood or old age. It should be noted that aging is the most common cause of sensorineural hearing loss followed by noise-induced hearing loss, and both are closely related to the formation of reactive oxygen species. Dietary antioxidant supplementation has been employed as a therapeutic strategy to prevent and/or delay the risks of major human diseases.

**Objective:**

To assess randomized clinical trials to determine the effect of antioxidant supplementation on the auditory thresholds in patients of different age groups with sensorineural hearing loss.

**Methods:**

This systematic review consisted of a search in the following databases: MEDLINE, CENTRAL, ScienceDirect, Scopus, Web of Science, LILACS, SciELO and ClinicalTrials.gov. Additionally, the gray literature was also searched. The search strategy included terms related to the intervention (antioxidant supplementation), primary outcome (sensorineural hearing loss), as well as terms related to randomized clinical trials to improve search sensitivity.

**Results:**

Based on 977 potentially relevant records identified through the search in the databases, ten full-text publications were retrieved for further evaluation. The increase in threshold at the 4 kHz frequency was statistically higher in the control group (1.89 [1.01–2.78], *p* < 0.0001) when compared to the NAC group and the ginseng group, whereas at 6 kHz, the threshold increase was higher in the control group (1.42 [−1.14–3.97], *p* = 0.28), but no statistically significant differences were found between groups.

**Conclusion:**

Ginseng was the antioxidant agent that showed the best effect in preventing auditory threshold worsening at the frequency of 4 kHz, but not at 6 kHz in patients with sensorineural hearing loss caused by exposure to high sound pressure levels. There was no improvement in the thresholds with vitamin E supplementation.

## Resumo

**Introdução:**

A perda auditiva é conceituada como qualquer diminuição da capacidade de ouvir e/ou detectar sons da fala ou do ambiente, independente da causa, tipo ou grau e pode ocorrer em diversos estágios da vida, durante a gestação ou parto, na infância, vida adulta ou na terceira idade. Convém destacar que o envelhecimento é a primeira causa de perda auditiva do tipo sensorioneural e em segundo lugar a perda auditiva induzida pelo ruído, ambas estão intimamente relacionadas com a formação de espécies reativas de oxigênio. Evidências têm sido acumuladas indicando que a suplementação com antioxidantes via alimentação tornou-se estratégia terapêutica para prevenir e/ou retardar os riscos das principais doenças humanas.

**Objetivo:**

Avaliar ensaios clínicos aleatórios para determinar qual o efeito da suplementação com antioxidantes sobre o limiar auditivo na perda auditiva sensorioneural em pacientes de diversas faixas etárias.

**Método:**

A formulação desta revisão sistemática consistiu na busca dos estudos nas seguintes bases de dados: MEDLINE, CENTRAL, *ScienceDirect*, *Scopus*, *Web of Science*, LILACS, SciELO e ClinicalTrials.gov. Adicionalmente, a literatura cinzenta também foi pesquisada. A estratégia de busca incluiu termos relacionados à intervenção (suplementação de antioxidantes), o desfecho primário (perda auditiva sensorioneural), bem como termos relacionados aos ensaios clínicos randomizados para melhorar e a sensibilidade da busca.

**Resultados:**

A partir de 977 registros potencialmente relevantes identificados através da busca nas bases de dados, dez publicações em texto completo foram recuperadas para avaliação mais aprofundada. O aumento no limiar na frequência de 4 kHz foi estatisticamente maior no Grupo Controle (1,89 [1,01–2,78], *p* < 0,0001) quando comparados com o grupo NAC e o Grupo Ginseng, já na frequência de 6 kHz o aumento no limiar foi maior no Grupo Controle (1,42 [–1,14–3,97], *p* = 0,28), porém, não foram encontradas diferenças estatisticamente significativas entre grupos.

**Conclusão:**

O Ginseng foi o antioxidante que evitou a piora do limiar auditivo na frequência de 4 kHz, mas não em 6 kHz, em pacientes com perda auditiva sensorioneural causada por exposição a elevados níveis de pressão sonora. Não foi observada melhora nos limiares com a suplementação com Vitamina E.

## Introduction

The integrity of the auditory system is important for an individual's communication and social interaction.^1^ Currently, deafness is considered a public health problem because of its high prevalence, and especially because of the adverse consequences it can have on intellectual, social, linguistic, cognitive, emotional and cultural aspects of human development.^1^, ^2^, ^3^, ^4^ Approximately 9.7 million Brazilians have hearing impairment and it is estimated that 360 million people worldwide have this health problem.^5^

Hearing loss is described as any impairment of the ability to hear and/or detect speech or environment sounds, regardless of cause, type or degree, and may occur at different stages of life; during pregnancy or childbirth, in childhood, adulthood or old age.^6^ According to Lloyd and Kaplan,^7^ it can be classified as mild, moderate, moderately severe, severe or profound, and may affect one or both ears; it may be classified as conductive, sensorineural and/or mixed^8^ and its origin can be congenital or acquired.^6^

The main causes of congenital hearing loss are genetic, congenital infections and use of ototoxic drugs during pregnancy. Acquired hearing loss can result from several factors including agenetic predisposition, meningitis sequelae, exposure to noise, aging, and the use of ototoxic drugs; in some situations, the etiology remains unknown.^9^

Aging is the most prevalent cause of sensorineural hearing loss, followed by noise-induced hearing loss as the second most frequent cause, and both are closely linked to the formation of reactive oxygen species (ROS), which are responsible for several types of damage to biological molecules present in the cochlea, as well as for the development of several other human diseases. In view of this, it would be desirable to be able to inactivate or reduce the formation of these free radicals, for example, by the use of antioxidants, agents that can inhibit or reduce damage in cells caused by free radicals.^10^

Foods, especially fruits, vegetables and legumes, also contain antioxidants, such as Vitamins C, E and A, chlorophyllin, flavonoids, carotenoids, curcumin, and others that can restrict the dissemination of chain reactions and free radical-induced damage.^11^, ^12^, ^13^ Evidence has accumulated indicating that dietary antioxidant supplementation has become a therapeutic strategy to prevent and/or to delay the risks of the main human diseases.^10^

Thus, the present meta-analysis aimed to evaluate randomized clinical trials to determine the effect of antioxidant supplementation on the auditory threshold in sensorineural hearing loss in patients of different age groups.

## Methods

This systematic review sought to answer the following question: Have patients of different ages with sensorineural hearing loss who received supplementation with antioxidants exhibited improved auditory thresholds? The present meta-analysis is reported according to the Preferred Reporting Items for Systematic Reviews and Meta-Analyses (PRISMA) Statement.^14^ The protocol was previously published in the PROSPERO database (http://www.crd.york.ac.uk/PROSPERO), (http://www.crd.york.ac.uk/PROSPERO), under n. CRD42015027677. Following the PRISMA criteria, the questions addressed in the objective refer to the PICO strategy, with patients being those of different ages with sensorineural hearing loss, while the intervention corresponds to the supplementation with antioxidants compared with subjects who did not receive supplementation. The results are related to auditory thresholds and the design of the assessed studies was the randomized clinical trial.

### Search strategy

The following databases were searched until June 2016: MEDLINE, CENTRAL, ScienceDirect, Scopus, Web of Science, LILACS, SciELO and ClinicalTrials.gov. Additionally, the following gray literature databases were also searched: OpenGrey.eu, DissOnline.de, NYAM.org and ClinicalEvidence.com. There was no manual search of the included articles and experts in the area were not contacted to avoid the risk of bias.^15^ The search strategy included terms related to the intervention (antioxidant supplementation), primary outcome (sensorineural hearing loss), as well as terms related to randomized clinical trials to improve search sensitivity.^16^ These descriptors were used in English for the search in most databases; however, the articles needed to have at least the title and/or abstract in English to be included in the present selection. The complete search strategy is shown in the supplemental material (Appendix 1). The search was not restricted to any year of publication or language.

### Eligibility criteria

Only randomized clinical trials that met the following criteria were included: 1) individuals who were diagnosed with sensorineural hearing loss (induced by ototoxic substances, such as cisplatin, gentamicin, amikacin, kanamycin, neomycin and others, noise-induced and presbycusis); 2) presence of a group submitted to supplementation with antioxidants through oral route (Vitamin C, Vitamin E, carotenoids, flavonoids and others) and a control group. There were no restrictions regarding gender, ethnicity or comorbidities. At the least, the studies needed to have assessed the auditory threshold as a result and to report the mean values found or the differences between the mean values.

The exclusion criteria were: 1) studies where dietary intervention was not related to hearing loss evaluation; 2) duplicate publications.

### Data extraction

The titles and abstracts of the retrieved articles were independently assessed by two investigators who were not blinded to the authors or titles of journals. The full versions of potentially eligible articles were retrieved for further assessment.

The primary outcome investigated in the studies was the auditory threshold improvement after antioxidant supplementation, after previous identification of the type of hearing loss verified and the evaluation tool used. Additionally, factors associated with the use of antioxidants, such as the type of substance used, the amount of antioxidant intake and the time of supplementation, were assessed as a secondary outcome.

All necessary information was extracted from the published articles, protocols and comments related to each study and where necessary, the authors were contacted for additional information. For studies that had more than two experimental groups, the most appropriate for the proposed objective was chosen by the authors. Any disagreements were resolved by consensus. In cases where there was no consensus, a third author was asked to make the final decision.

In addition to the outcome data, we also extracted the authors’ names, study title, year of publication, country, age range of the groups, number of subjects in each group, type and degree of hearing loss per ear. A standard form for data storage was created based on the model adopted by Cochrane.^17^

### Assessment of the risk of bias

The risk of bias was analyzed according to the recommendations of the Cochrane Handbook^18^ at the primary outcome level.

The quality of the studies was evaluated by two independent researchers in five categories: generation of the appropriate sequence; allocation concealment; blinding of the participants, the researcher and the evaluators; handling of missing data for subsequent final judgment.

### Data analysis

The auditory threshold improvement after antioxidant supplementation was analyzed. The effects of the treatment through the assays were combined, and the weighted mean difference for the outcome measures was calculated.

For this, a random effects model was used as a measure of the effect of the mean difference between the groups and as a statistical method of analysis. An *α* value <0.05 was considered statistically significant. When it was not possible to obtain adequate data for analysis, the Cochrane recommendations were followed.

Statistical heterogeneity between the studies was tested using the Cochran test and inconsistency was tested using the *I*^2^ test. A value of *p* < 0.10 was considered statistically significant. When necessary, study characteristics considered as potential sources of heterogeneity were included in a subgroup analysis. In addition, in case of heterogeneity, studies were removed, one by one, to investigate whether the study removed was the source of heterogeneity.

All analyses were performed using the RevMan 5.3 software (Cochran Collaboration).

## Results

### Included studies

**Figure 1:**
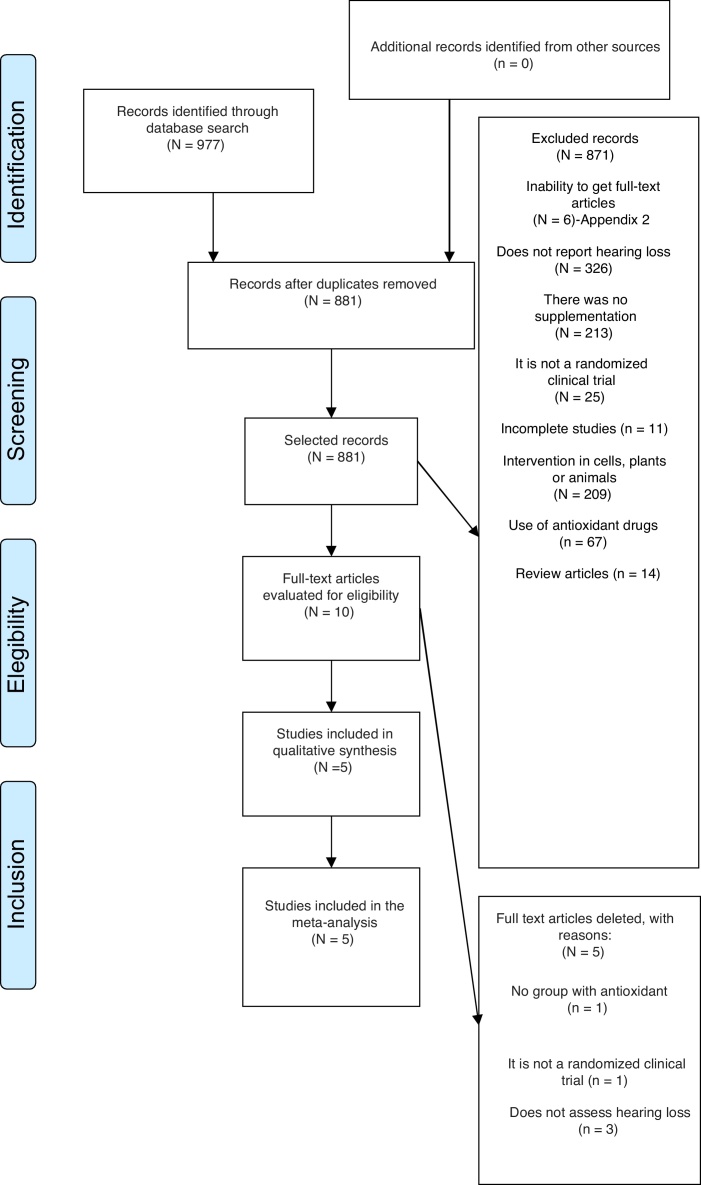
Flow diagram of study selection.

Based on 977 potentially relevant records identified through the search in the databases, ten full-text publications were retrieved for further evaluation. Of these, five were excluded after the full-text analysis, five articles were included in the qualitative analysis and of these, four in the quantitative analysis (Table 1). The flow chart showing study search and selection is shown in Fig. 1.

**Table 1:** General characteristics of included studies.

Source	City (country)	Sample (gender)	Mean age (years) ± standard deviation
Joachims et al., 2003^27^	Haifa (Israel)	66 patients	41
		33 in S group	15–70 years (mean age: 41 years)
		33 in C group	
Kharkeli et al., 2007^28^	Georgia (USA)	52 patients (23 males; 29 females)	32.6 (±11.1) – experimental group
		Vitamin E: 23	29.0 (±10.3) – control group
		Placebo: 29	
Polanski and Cruz 2013^26^	São Paulo (Brazil)	120 patients (53 males; 67 females)	74.3 ± 8.5 – ginkgo biloba group
			74.6 ± 5.4 – α-lipoic acid + Vitamin C group
		Placebo: 30	
		Ginkgo biloba: 30	
		30 α-lipoic acid + Vitamin C group	71.2 ± 6.4 – papaverine hydrochloride + Vitamin E group
		30 papaverine hydrochloride + Vitamin E group	75.4 ± 7.0 – placebo group
Doosti et al., 2014^24^	Teheran (Iran)	48 workers (male)	39.12 (±5)
		NAC – 16	39.38 ± 6.2 – NAC group
		Ginseng – 16	38.38 ± 4.4 – ginseng group
		Control – 16	39.62 ± 4.6 – control group
Doosti et al., 2014^25^	Teheran (Iran)	48 workers (male)	39.12 (± 5)
		NAC – 16	39.38 ± 6 – NAC group
		Ginseng – 16	38.38 ± 4 – ginseng group
		Control – 16	39.62 ± 4 – control group

NAC, N-acetyl-cysteine.

Among the five excluded studies, the work of Kaya et al.^19^ although it was included to have the eligibility criteria verified, was subsequently excluded due to the choice of whether or not to receive antioxidant supplementation was at the patient's discretion, not being performed in a randomized manner, which does not characterize a randomized clinical trial.

The study by Schmitz et al.^20^ evaluated the risk of hearing loss and not the hearing loss itself, and was thus different from the objectives of the other studies included in this review and, therefore was excluded as well. The studies by Gilles et al.^21^ and Quaranta et al.^22^ were eliminated because they assessed temporary hearing loss. Finally, the study by Reisser and Weidauer^23^ was also not included because the group of individuals who received the antioxidants used the infusion, and not the supplementation technique, which corresponds to the intervention of the present study. Table 1 shows the general characteristics of the included articles.

Table 2 depicts the characteristics of the performed intervention and the outcomes found by the articles included in this systematic review.

**Table 2:** Characteristics of the included studies regarding the intervention and the results found.

Source	Type of hearing loss observed	Intervention for hearing loss	Antioxidant used	Amount administered	Time of supplementation	Hearing loss assessment tool	Effect on auditory threshold	Results found	Observations
Joachims et al., 2003^27^	Sudden hearing loss (<7 days)	Group C: prednisone (1 mg/kg/day); magnesium sulfate intravenous (4 g/day); carbogen through mask (95% O_2_ + 5% CO_2_) – 30 min/4 × /day	Vitamin E	Grupo S – 400 mg (2 × /day)	a	Pure-tone and vocal audiometry.	Group C – 45.45% (*n* = 15) showed the result	Improvement of 75% or more in the recovery rate	Vitamin E was found as being beneficial in the treatment of sudden hearing loss
								Group C – improved N: 15 Total N: 33	
		Group S: intervention of group C + Vitamin E (400 mg 2 × /day).					Group S – 78.78% (*n* = 26) showed the result	Group S – improved N: 26 Total N: 33	
Kharkeli et al., 2007^28^	Induced by ototoxic substances.	Experimental group – 80 mg gentamicin (3×/day) + Vitamin E (2800 mg/day in three doses: 1200 mg, 800 mg, 800 mg)	[1,0]Vitamin E	[1,0]2800 mg/day in three doses: 1200 mg; 800 mg; 800 mg)	[1,0]7 days	[1,0]Pre- and post-pure-tone audiometry	[1,0]Auditory thresholds increased in a similar number of patients from both groups, but with no statistical difference	The analyzed criteria showed no statistical difference	[1,0]Vitamin E is not clinically effective against gentamicin-induced ototoxicity. However, due to the limited number of subjects, the conclusion should be considered delicate
								Control group Improved N: 26 Total N: 29	
		Control group – 80 mg gentamicin (3×/day) + placebo (peanut oil, gelatin, glycerin and sorbitol)						Experimental group Improved N: 20 Total N: 23	
Polanski and Cruz, 2013^26^	Presbycusis.	Group 1 – ginkgo biloba (120 mg/day)	Ginkgo biloba; α-lipoic acid + Vitamin C; Vitamin E.	Ginkgo biloba (120 mg/day); α-lipoic acid (60 mg/day) + Vitamin C (600 mg/day); Vitamin E (400 mg/day)	6 months	Pre- and post-pure-tone and vocal audiometry	There were differences in the audiological thresholds: 500 Hz, 1000 Hz and 8000 Hz, being greater for group 4 when compared to group 2	The results before and after treatment were not significantly different in any treatment group	The results did not show a statistically significant difference of the effects of antioxidant substances on the auditory thresholds of this population during the 6-month study period
		Group 2 – α-lipoic acid (60 mg/day) + vitamin C (600 mg/day)							
		Group 3 – papaverine hydrochloride (100 mg/day) + vitamin E (400 mg/day)							
		Group 4 – placebo.							
Doosti et al., 2014^24^	Noise-induced hearing loss.	Control group – no intervention	Ginseng	Ginseng group – 200 mg/day	14 days (2 weeks)	Distortion product otoacoustic emissions – DPOAE (1, 2, 4 and 6 kHz)	NAC and ginseng groups showed the result; additionally, the NAC group showed better amplitude of DPOAE than the ginseng group	Reduced DPOAE amplitude at high frequencies (4 and 6 kHz) in both ears	The generalization of the results found in relation to the protective effects of these interventions requires assays with different doses and in a larger population
		NAC group – NAC 1200 mg/day						Control group N improved: Total N: 16	
		Ginseng group– 200 mg/day.						NAC group N improved: Total N: 16	
								Ginseng group N improved: Total N: 16	
Doosti et al., 2014^25^	Noise-induced hearing loss	Control group – no intervention;	Ginseng	Group G – ginseng 200 mg/day	14 days (2 weeks)	Pre- and post-pure-tone audiometry and high-frequency audiometry	Groups N and G showed the result	Reduced the temporary change in auditory threshold (4, 6 and 16 kHz)	NAC and ginseng showed to have preventive effects of NIHL. This beneficial effect was most often seen in Group N
		Group N – NAC 1200 mg/day					Group N showed greater reduction	Control group Improved N: Total N: 16	
		Group G –ginseng 200 mg/day.						Group NAC Improved N: Total N: 16	
								Ginseng group Improved N: Total N: 16	

NAC, N-acetyl-cysteine.

a Not specified.

### Assessment of the risk of bias

The risk of bias in studies at the primary outcome level is shown in Table 3. Of the five included studies, three did not report the method used for sequence generation; regarding allocation concealment, all articles do not provide sufficient information on this process to allow judgment. All five articles reported the blinding of the evaluators, as well as the justification for missing data, when they occurred.

**Table 3:** Risks of bias of included articles.

Source	Sequence generation	Allocation concealment	Participant and researcher blinding	Blinding of evaluators	Handling of missing data
Joachims et al., 2003^27^	Uncertain	Uncertain	Low	Low	Low
Kharkeli et al., 2007^28^	Uncertain	Uncertain	Low	Uncertain	Low
Polanski & Cruz, 2013^26^	Low	Low	Low	Low	Low
Doosti et al., 2014^24^	Low	Low	Low	Low	Low
Doosti et al., 2014^25^	Low	Low	Low	Low	Low

### Data analysis

**Figure 2:**
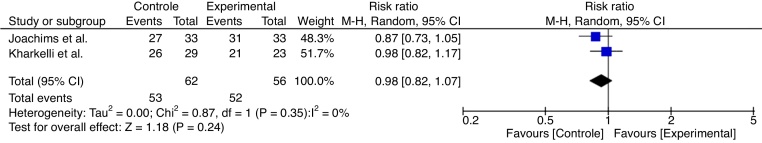
Overall effect of antioxidant supplementation on the auditory threshold.

**Figure 3:**
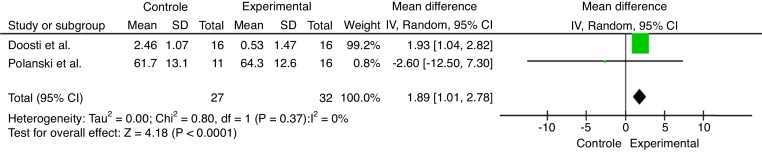
Overall effect of antioxidant supplementation on auditory threshold at the 4 kHz frequency.

**Figure 4:**
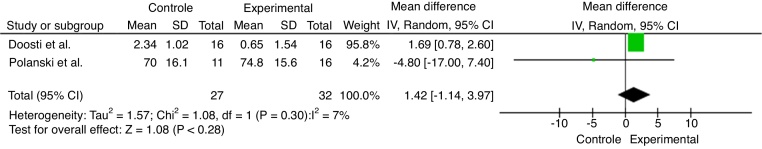
Overall effect of antioxidant supplementation on the auditory threshold at the frequency of 6 kHz.

When considering the diversity of the objectives and methodologies of the selected articles, quantitative data analyses were performed by combining articles that showed results in common. Fig. 2 exhibits the studies that showed auditory threshold improvement after antioxidant supplementation. Figure 3, Figure 4 show the overall effect of antioxidant supplementation on the auditory threshold at the specific frequencies of 4 kHz and 6 kHz, respectively.

Although Doosti et al.^24^ demonstrated improvement in the amplitude of DPOAE at high frequencies (4 and 6 kHz) in both ears indicating improvement in hair cell function, they did not present enough data to document auditory threshold improvement/worsening after the use of antioxidants, or data that allowed comparison with those from the other studies, by Doosti et al.^25^ and Polanski and Cruz,^26^ with respect to any effects on the specific frequencies of 4 and 6 kHz.

Although the control group showed a higher risk of auditory threshold worsening (RR = 0.93 [0.82–1.05], *p* = 0.24), there were no statistically significant differences among the groups.

Threshold increase was statistically higher in the control group (1.89 [1.01–2.78]; *p* < 0.0001) when compared to the NAC group and the ginseng group.

Threshold increase was higher in the control group (1.42 [−1.14–3.97], *p* = 0.28); however, no statistically significant differences were found between groups.

From the meta-analyses performed in the aforementioned studies, as shown in Fig. 2, it can be verified that the selected studies sought to verify the recovery rate – treatment improvement^27^, ^28^ and in Figure 3, Figure 4, the temporary change in the auditory threshold (at frequencies of 4 and 6 kHz).^25^, ^26^ Although the results showed a possible general improvement in the auditory threshold of the experimental group,^27^, ^28^ in addition to an increase in the auditory threshold for the frequency of 6 kHz in the experimental group,^25^, ^26^ the meta-analysis performed did not show significant differences between groups. However, at the 4-kHz frequency, the auditory threshold worsening was significantly lower in the experimental group when compared to the control group.^25^, ^26^

## Discussion

The four studies included in the quantitative analysis corresponded to a total of 286 individuals, randomly assigned either to a group that received antioxidant supplementation or one that did not receive it (control group). The four studies had, in addition to antioxidant supplementation, the use of concomitant drugs, with the intervention for supplementation being chosen, and one study had more than two intervention groups; the groups that better fit the analysis were determined by consensus.

The studies^24^, ^25^, ^26^, ^27^, ^28^ evaluated different types of hearing loss and, therefore, used different interventions to assess the effect of antioxidant supplementation on their respective types of loss. Moreover, the studies differed regarding the antioxidant supplementation, dose used and time of supplementation. The tools used for hearing loss evaluation were: pure-tone and speech audiometry,^27^ distortion product otoacoustic emissions,^24^ pre- and post-pure tone and high-frequency pure-tone audiometry^25^ and pre- and post-pure tone audiometry^26^, ^28^ and therefore, the results were different among the studies.

The study by Joachims^27^ investigated the possible benefits of the antioxidant effect of Vitamin E in the treatment of sudden deafness. The study design consisted of dividing the participants into two groups, Group C consisting of patients receiving prednisone (1 mg/kg/day), intravenous magnesium sulfate (4 g/day) and carbogen through a face mask (95% O_2_ + 5% CO_2_) for 30 min four times daily, whereas Group S received Vitamin E (400 mg twice daily) in addition to the Group C intervention. The recovery rate, calculated as the hearing gain divided by the hearing level difference between the affected individuals and affected ears, was greater than 75% in 41 of the 66 (62.12%) patients included in the study. This rate was reached in 26 (78.78%) patients in Group S (treated with Vitamin E), compared with 15 (45.45%) patients in the control group (Group C). There was improvement in patients treated with the addition of Vitamin E, but the authors recommended further studies to better understand the role of antioxidants in sudden deafness.

Aiming to assess the otoprotective effect of Vitamin E on the ototoxic effect of gentamicin, Kharkeli et al.^28^ randomly assigned 52 patients receiving gentamicin to treat acute pulmonary infection to two different groups, an experimental group, receiving 80 mg of gentamicin (three times/day) associated with 2800 mg/day of Vitamin E, divided into three doses (1200 mg, 800 mg, 800 mg), and a control group that also received 80 mg of gentamicin (three times/day) along with a placebo, consisting of peanut oil, gelatin, glycerin and sorbitol. The duration of the experiment was 7 days and pure-tone audiometry results analysis, before beginning and 6–8 weeks after completion of the supplementation were compared. An increase in auditory threshold was observed in both groups, but without statistical significance, suggesting that Vitamin E does not have an otoprotective effect on the ototoxic effect exerted by gentamicin.

These two studies were the only ones that showed the “*n*” of participants of each study group (control group and the intervention group, that received Vitamin E) as well as their respective percentages of improvement. Although both studied the effect of the same antioxidant on sensorineural hearing loss, the causes of this loss were different in each of the studies. Whereas in the study by Joachims,^27^ sensorineural hearing loss was of sudden origin and evaluated unilaterally and suggested a possible improvement with Vitamin E supplementation, the study by Kharkeli et al.,^28^ studied gentamicin-induced sensorineural hearing loss, one of the main ototoxic drugs used for the treatment of pulmonary infection, and the use of the same vitamin did not show the otoprotective function.

Considering that few articles were found using antioxidant supplementation aiming to evaluate auditory protection and improvement, and that the studies found were not grouped in the same category, with respect to the studied antioxidant or the cause of the sensorineural hearing loss, as well as small sample sizes, the studies were considered fragile and signaled a need to carry out other studies on this topic.

Polanski and Cruz^26^ sought to evaluate the effect of antioxidant agents on the hearing threshold of patients with presbycusis. For that purpose, the sample (*n* = 120) was divided into four groups that received the following treatments: dry extract of ginkgo biloba (120 mg/day), α-lipoic acid (60 mg/day) and vitamin C (600 mg/day), papaverine hydrochloride (100 mg/day) and Vitamin E (400 mg/day) or placebo. All participants were assessed at enrollment and after 6 months, using pure-tone audiometry thresholds (by means and isolated frequencies) and the percentage of the Speech Recognition Index (SRI). The results revealed no statistically significant changes in the auditory thresholds after the treatments during the study period.

The study by Doosti et al.^25^ aimed to verify the otoprotective effect of N-acetyl-cysteine (NAC) and ginseng in workers exposed to high sound pressure indexes, i.e., noise-induced hearing loss (NIHL).

The authors randomly separated 48 workers exposed to continuous noise in a textile factory in three groups: 1) control group (*n* = 16) corresponding to those who did not receive antioxidant drugs; 2) NAC group (*n* = 16) that received oral N-acetyl-cysteine (1200 mg/day) and; 3) ginseng group (*n* = 16) who received the oral antioxidant (200 mg/day). Pure-tone and high-frequency audiometries were performed on the first day and 15 days after the intervention. The results showed a temporary improvement in the noise-induced thresholds for the NAC Group and the ginseng group at the 4, 6 and 16 kHz frequencies (*p* < 0.001) in both ears. Moreover, the otoprotective effects were higher in the NAC Group. Thus, the study shows that NAC and ginseng can reduce the impact of occupational noise to which workers are exposed and recommend further studies to prove the benefits of antioxidant use in hearing conservation programs.

The studies by Polanski and Cruz^26^ and Doosti et al.^25^ used audiometry as a tool to evaluate hearing loss and, despite using different antioxidants, they analyzed similar audiometric frequencies, such as 4 and 6 kHz. The meta-analysis verified that at 6 kHz, no improvement was observed with the use of antioxidants, perhaps because of insufficient time of exposure/use of the antioxidant for this to occur. However, due to the evaluation technique used, it is not possible to state that there was no improvement in hair cell function at that frequency. To prove that, a more detailed auditory evaluation would be necessary, with distortion product otoacoustic emissions analysis.

On the other hand, the meta-analysis of the 4 kHz frequency showed significant results with the use of antioxidants, when NIHL was the cause of sensorineural hearing loss and ginseng the antioxidant responsible for the otoprotective effect. These results are similar to those found by Doosti et al.,^25^ since it accounts for 99.2% of the weight in the meta-analysis (Fig. 3).

Exposure to intense noise over time activates physical, morphological and mechanical mechanisms that can cause cochlear damage and result in hearing loss.^29^, ^30^ Additionally, molecular and metabolic mechanisms may also be responsible for this type of lesion.^31^, ^32^

Such lesions may be temporary or permanent.^25^ The mechanisms of permanent hearing loss of cochlear origin are caused by the death of hair cells (internal and external), primary afferent neurons, or both.^33^ The exact mechanism of temporary hearing loss is unclear.^34^ Recent studies indicate that the formation of reactive oxygen species (ROS) and oxidative stress are the main metabolic causes of temporary hearing loss.^33^, ^34^

ROS are molecules characterized by the presence of an unpaired electron and are naturally present in the body, participating in homeostasis and in important signaling pathways. However, as a consequence of the endogenous antioxidant system imbalance, ROS levels can increase to toxic levels and cause cell death from damage to membranes, cytosol and mitochondria.^35^, ^36^

Specifically, the excess of free radicals in the cochlear sensory epithelium, spiral ganglion neurons and in the cells of the cochlea vascular stria may play a relevant role in the development of hearing loss.^36^ Excess ROS is clearly the key factor in the pathogenesis of other stress- and age-induced otologic conditions, and also in hearing loss due to exposure to intense noise and the effect of ototoxic drugs.^37^, ^38^

It is known that NAC is a potent free-radical scavenger and a precursor of glutathione (GSH), one of the main antioxidant enzymes, which can neutralize the noise effects^39^ and increase GSH production.^40^ Some studies have shown a positive effect of NAC on permanent hearing loss.^37^, ^39^ The study by Lin et al. (2010)^41^ showed that oral administration of 1200 mg/day of NAC for 14 days reduced the temporary hearing loss induced by noise at the frequencies of 3, 4 and 6 kHz. Ginseng has important antioxidant and anti-apoptotic properties and, consequently, may play an important role in NIHL.^40^

The fact that the antioxidant agent delayed hearing threshold worsening at the specific frequency of 4 kHz may have been determined because that specific group of cells was the most activated during exposure to loud noises. This activation occurs through the three main mechanism of action: 1) resonance of the external auditory canal that occurs, in average, at the frequency of 3.8 kHz and the auditory pavilion around 5 kHz; 2) non-linear, energy transmission through the middle ear, especially in the tympanic membrane where it is most efficient at the frequencies of 1–5.5 kHz; 3) acoustic reflex only attenuates intense sounds that have frequencies below 2 kHz, and is most efficient below 1 kHz.^42^ There may be other unknown physiological mechanisms in the cochlea and the auditory pathway to account for this observed effect at 4 kHz. Thus, a greater activation of this region would result in increased cellular metabolism and, consequently, an opportunity to benefit from the antioxidants present in ginseng.

The use of ginseng attenuated the hydrogen peroxide-induced oxidative stress and apoptosis in human neuroblastoma cells, but only animal studies were carried out to observe the effect of ginseng on NIHL.^25^, ^43^ Although it has been suggested that ginseng is effective in preventing hearing damage in patients exposed to intense noise, there are no randomized clinical trials to test its effect on NIHL.^25^, ^44^

In view of the results found, it is suggested that antioxidants may have otoprotective effects by reducing the harmful effects of reactive oxygen species on the cochlea and, consequently, on sensorineural hearing loss.

## Conclusion

Ginseng was the antioxidant that prevented auditory threshold worsening in the 4-kHz but not at the 6-kHz frequency in patients with sensorineural hearing loss caused by exposure to high levels of sound pressure. There was no improvement in the thresholds with vitamin E supplementation.

## Conflicts of interest

The authors declare no conflicts of interest.
