# Supplementary material for: Effect of antioxidant supplementation on the auditory threshold in sensorineural hearing loss: a meta-analysis
Source: Braz J Otorhinolaryngol. 2017 Aug 26;84(3):368–80. doi: 10.1016/j.bjorl.2017.07.011 (PMC9449190; doi:10.1016/j.bjorl.2017.07.011)
Supplement: Supplementary file 1 [file mmc1.docx]

| **Appendix 1 Search strategies used in databases.** |
| --- |
|  |
| **MEDLINE (via PubMed)** |
| **1.** ((((((("antioxidants"[Pharmacological Action] OR "antioxidants"[MeSH Terms] OR "antioxidants"[All Fields]) OR anti-oxidant[All Fields]) OR "Vitamin E"[Mesh]) OR ("ascorbic acid"[MeSH Terms] OR vitamin C[Text Word])) OR ("carotenoids"[MeSH Terms] OR carotenoids[Text Word])) OR ("flavonoids"[MeSH Terms] OR flavonoids[Text Word])) OR ("glutathione"[MeSH Terms] OR glutathione[Text Word])) OR ("cysteine"[MeSH Terms] OR cysteine[Text Word]) |
| **2.** ((("Hearing Loss"[Mesh] OR "Hearing Loss, Noise-Induced"[Mesh]) OR "Hearing Loss, Sensorineural"[Mesh]) OR ototoxicity[All Fields] |
| **3.** (randomized controlled trial [pt] OR controlled clinical trial [pt] OR randomized controlled trials [mh] OR random allocation [mh] OR double-blind method [mh] OR single-blind method [mh] OR clinical trial [pt] OR clinical trials [mh] OR (“clinical trial” [tw]) OR ((singl* [tw] OR doubl* [tw] OR trebl* [tw] OR tripl* [tw]) AND (mask* [tw] OR blind* [tw])) OR (“latin square” [tw]) OR placebos [mh] OR placebo* [tw] OR random* [tw] OR research design [mh:noexp] OR follow-up studies [mh] OR prospective studies [mh] OR cross-over studies [mh] OR control* [tw] OR prospectiv* [tw] OR volunteer* [tw]) NOT (animal [mh] NOT human [mh]) |
| **4.** #1 AND #2 AND #3 |
|  |
| **Cochrane Clinical Trials** |
| (antioxidants OR “vitamin e” OR “ascorbic acid” OR carotenoids OR flavonoids OR cysteine OR glutathione) AND (“hearing loss” OR “hearing loss, noise-induced” OR “hearing loss, sensorineural” OR presbycusis OR ototoxicity): ti,ab,kw |
|  |
| **ScienceDirect** |
| (antioxidants OR “vitamin e” OR “ascorbic acid” OR carotenoids OR flavonoids OR cysteine OR glutathione) AND (“hearing loss” OR “hearing loss, noise-induced” OR “hearing loss, sensorineural” OR presbycusis OR ototoxicity) |
|  |
| **ClinicalTrials.gov** |
| (antioxidants OR “vitamin e” OR “ascorbic acid” OR carotenoids OR flavonoids OR cysteine OR glutathione) AND (“hearing loss” OR “hearing loss, noise-induced” OR “hearing loss, sensorineural” OR presbycusis OR ototoxicity)\| Closed Studies \| Interventional Studies |
|  |
| **LiLACS** |
| antioxidantes AND “perda auditiva” |
|  |
| **Scopus** |
| (TITLE-ABS-KEY((antioxidants OR “vitamin e”OR “ascorbic acid”OR carotenoids OR flavonoids OR cysteine OR glutathione)) AND TITLE-ABS-KEY((“hearing loss”OR “hearing loss, noise-induced”OR “hearing loss, sensorineural”OR presbycusis OR ototoxicity))) AND (“randomized controlled trial”OR “controlled clinical trial”OR “randomized clinical trial”OR “randomized trial”)AND DOCTYPE(ar) OR DOCTYPE(cp) OR DOCTYPE(no) OR DOCTYPE(sh) |
|  |
| **Web of Science** |
| Title: ((antioxidants OR vitamin e OR ascorbic acid OR carotenoids OR flavonoids OR cysteine OR glutathione) AND (hearing loss OR hearing loss, noise-induced OR hearing loss, sensorineural OR presbycusis OR ototoxicity)) |
|  |
| Filter: No patent |
|  |
| **Circumpolar Health Bibliographic Database; SciELO;The New York Academy of Medicine, ClinicalEvidence.com** |
| (antioxidants OR “Vitamin E”OR “ascorbic acid”OR carotenoids OR flavonoids OR cysteine OR glutathione) AND (“hearing loss”OR “hearing loss, noise-induced”OR “hearing loss, sensorineural”OR presbycusis OR ototoxicity)AND (“randomized controlled trial”OR “controlled clinical trial”OR “randomized clinical trial”OR “randomized trial” ) |
|  |
| **OPENGREY.EU** |
| (antioxidants OR “Vitamin E”OR “ascorbic acid”OR carotenoids OR flavonoids OR cysteine OR glutathione) AND (“hearing loss”OR “hearing loss, noise-induced”OR “hearing loss, sensorineural”OR presbycusis OR ototoxicity) AND (“randomized controlled trial”OR “controlled clinical trial”OR “randomized clinical trial”OR “randomized trial”) |
|  |
| **DissOnline.de “**Antioxidantien”AND Hörverlust” |

| **Appendix 2 Full texts excluded from the analysis.** | | |
| --- | --- | --- |
| **Source** | **Title** | **Reason** |
| Romeo; Giorgetti | Therapeutic effects of Vitamin A associated with vitamin E in perceptual hearing loss | 1985 article. |
| Nestrugina | Effect of supplementary ingestion of Vitamins B1 and C on the function of the auditory organ in workers of “noisy” trades | 1969 article. |
| Creston;Gillespie, [Larson Al](http://www.ncbi.nlm.nih.gov/pubmed/?term=Larson%20AL%5BAuthor%5D&cauthor=true&cauthor_uid=5339343) | [Bioflavonoid therapy in sensorineural hearing loss: A double-blind study.](http://www.ncbi.nlm.nih.gov/pubmed/5339343) | 1966 article. |
| Ivstam | [Vitamin A as a means of reducing industrial deafness.](http://www.ncbi.nlm.nih.gov/pubmed/13718332) | 1960 article. |
| Mann; Beck; Beck | Calcium antagonists in the treatment of sudden deafness. | 1986 article. |
| Gordin et al. | Antioxidants: a new armament in the treatment of idiopathic sudden hearing loss | 2004 article - Requested to the author by email, but without return. |
